# External Quality Assurance for Laboratory Identification and Capsular Typing of *Streptococcus pneumoniae*

**DOI:** 10.1038/s41598-017-13605-8

**Published:** 2017-10-16

**Authors:** Hans-Christian Slotved, Carmen L. Sheppard, Tine Dalby, Arie van der Ende, Norman K. Fry, Eva Morfeldt, Outi Nyholm, Assaf Rokney, Merav Ron, Lotta Siira, Kevin J. Scott, Andrew Smith, Louise Thom, Maija Toropainen, Didrik F. Vestrheim

**Affiliations:** 1 Department of Bacteria, Parasites and Fungi - Neisseria and Streptococcus Reference Laboratory, Statens Serum Institut, Copenhagen, Denmark; 2 Respiratory and Vaccine Preventable Bacteria Reference Unit, Public Health England–National Infection Service, London, UK; 3 Department of Medical Microbiology and the Netherlands Reference Laboratory for Bacterial Meningitis, Academic Medical Center, Amsterdam, The Netherlands; 4 Department of Microbiology, Laboratory Surveillance of Bacterial Pathogens, The Public Health Agency of Sweden, Solna, Sweden; 5 Department of Health Security, National Institute for Health and Welfare, Helsinki, Finland; 6 Government Central Laboratories, Ministry of Health, Jerusalem, Israel; 7 Scottish Haemophilus Legionella Meningococcus & Pneumococcus Reference Laboratory (SHLMPRL), New Lister Building, Glasgow Royal Infirmary, Glasgow, Scotland UK; 8 Department of vaccine preventable diseases, Norwegian Institute of Public Health, Oslo, Norway

## Abstract

An external quality assessment (EQA) scheme for pneumococcal serotype identification has been performed over a period of 11 years, by a network of European pneumococcal reference laboratories. We report the results from the EQA, and present an assessment of the acceptability and utility of the EQA scheme. Reports from 22 EQA panels distributed in 2005–2016 were analysed. Each EQA panel consisted of seven isolates. A questionnaire including seven questions related to the acceptability and utility of the EQA scheme was distributed to all participating laboratories. Altogether, 154 pneumococcal isolates were tested. Of the 92 serologically distinct serotypes currently defined, 49 serotypes were included in the rounds. Discrepant results were observed in eight EQA rounds, involving 11 isolates (7.1%, 95% CI: 4% to 12%). All participating laboratories reported that the EQA scheme was useful for quality assurance purposes. Our results show that comparable serotyping data can be obtained in different laboratories. The EQA participation helps to keep the typing procedures at a high standard and provides data for accreditation purposes. The EQA is helpful when new technologies are introduced, and reveal limitations of both genotypic and phenotypic methods. Continuation of the presented EQA scheme is planned.

## Introduction

*Streptococcus pneumoniae* (pneumococcal) infection is a cause of high morbidity and mortality among children and elderly worldwide, and one of the most frequent causes of bacteraemia and meningitis in infants globally^1–3^. It is essential to detect, identify and serotype *S. pneumoniae* correctly^4,5^ in order to provide reliable surveillance data to guide pneumococcal vaccination policies, and to evaluate the effect of national vaccination programs on pneumococcal serotype distribution^6^. Pneumococcal serotyping performed at national reference laboratories is an important part of Invasive Pneumococcal Disease (IPD) surveillance and pivotal for assessing the effectiveness of pneumococcal immunisation programmes introduced in many countries during the last 17 years^6,7^. The impact of the immunisation programs on the epidemiology of pneumococcal disease globally can be assessed by compilation and comparison of pneumococcal serotype data from different countries and surveillance sites^8^. When comparing data between different reference laboratories, serotyping results have to be comparable for the different laboratories, irrespective of the procedures and serotyping methodologies used^9,10^.

External quality assessment (EQA) schemes allow laboratories to assess their performance to identify and type a pathogen, even when different methodologies have been used^11–13^. The EQA results can also document that laboratory procedures at a certain time point are in accordance with internationally agreed procedures^9,10,14^.

Many methods have been described for serotyping of *S. pneumoniae* ^7,15^. The methods can be divided into two major types of identification; phenotypic and genotypic^7,12,15^.

EQA schemes for pneumococcal serotyping can aid laboratories in performing correct capsular identification of *S. pneumoniae*, despite the use of different methodologies. We describe an international EQA scheme that has been performed semiannually over a period of 11 years, based on a collaborative network of pneumococcal reference laboratories. Results from the scheme were summarised in order to evaluate the performance of pneumococcal reference laboratories using different serotyping procedures. We also assessed the acceptability and usefulness of the EQA scheme. Based on this evaluation, recommendations for setting up an EQA scheme were formulated.

## Materials and Methods

### Participants

The EQA scheme started in June 2005 with an agreement between the two reference laboratories SHLMPRL and SSI. Currently, eight institutions from eight countries (Table 1) participate in the EQA distributions: 1. Scottish Haemophilus, Legionella, Meningococcus & Pneumococcus Reference Laboratory (SHLMPRL), Scotland; 2. Statens Serum Institut (SSI), Denmark; 3. Norwegian Institute of Public Health (NIPH), Norway; 4. National Institute for Health and Welfare (THL), Finland; 5. Public Health England (PHE), England. 6. The Public Health Agency of Sweden (PHAS), Sweden; 7. Netherlands Reference Laboratory for Bacterial Meningitis (NRLBM), the Netherlands; and 8. National Reference Laboratory, Israel.

**Table 1:** Year of signing agreement for EQA programs.

Year	Institutions	Comments
2005	Scottish Haemophilus, Legionella, Meningococcus & Pneumococcus Reference Laboratory (**SHLMPRL**) Scotland and Statens Serum Institut (**SSI**) Denmark	This EQA was performed based on a personal agreement between the two laboratories on May 23, 2005.
2009	**SHLMPRL**, Norwegian Institute of Public Health (**NIPH**) Norway, **SSI**	Agreement of External Quality Assurance on serotyping of *Streptococcus pneumoniae*
2010	**SHLMPRL**, **NIPH**, **SSI**, National Institute for Health and Welfare (**THL**) Finland, Public Health England (**PHE**), England	Agreement of External Quality Assurance on serotyping of *Streptococcus pneumoniae*
2013	**SHLMPRL**, **NIPH**, **SSI**, **THL**, **PHE**	Agreement of External Quality Assurance on serotyping of *Streptococcus pneumoniae*
2015	**SHLMPRL**, **NIPH**, **SSI**, **THL**, **PHE**, The Public Health Agency of Sweden (**PHAS**), Sweden.	Agreement of External Quality Assurance on serotyping of *Streptococcus pneumoniae*
2017	**SHLMPRL**, **NIPH**, **SSI**, **THL**, **PHE**, **PHAS**, Netherlands Reference Laboratory for Bacterial Meningitis (**NRLBM**), the Netherlands, **National Reference Laboratory**, Israel	Agreement underway including NRLBM and National Reference Laboratory Israel

The names of several institutions have changed over the years; the most recent names and abbreviations have been used throughout the manuscript.

### Bacterial isolates and dispatches

The EQA panels were prepared and shipped by the sending laboratories selected on a rotating principle, in which all participating laboratories in turn became the “sending laboratory”.

Each EQA panel was shipped in compliance with international packaging and transportation requirements for infectious substances according to the International Air Transportation Association (IATA) Dangerous Goods Regulations UN3373 Category B (see http://www.iata.org). Each panel consisted of seven clinical *S. pneumoniae* isolates chosen by the sending laboratory. The isolates were sent blinded, but labelled with a unique panel number, in a suitable transportation medium and container at ambient temperature. The EQA results presented in this study are from EQA panels sent in the period from June 2005 to September 2016.

### EQA panels

The pneumococcal isolates were serotyped by each laboratory, using their routine procedures for pneumococcal serotyping. After species identification and serotyping, the results were returned to the sending laboratory by e-mail on completion of a submission form within 6 weeks. The receipt of both isolates and the results were confirmed by e-mail.

### Concluding and reporting of EQA results

The combined results were reported back by the sending laboratory to all participating reference laboratories. If all the results were in agreement, a final report was prepared by the sending laboratory after which the EQA round in question was concluded.

If discrepancies were found, this was reported to the laboratory sending the discrepant result and thereby giving the laboratory an opportunity to reanalyse the isolate or isolates in question after which the results were compared again. If the results were in agreement, the procedure above was followed. If there were still discrepancies, the isolates in question were sent again from the sending laboratory to be reanalysed in both laboratories. Based on this test, the sending laboratory and the laboratory with the discrepancies discussed the results obtained and tried to agree on a consensus result, including the test results, on which the majority of the laboratories reached. If needed an external laboratory was contacted to perform additional serotyping. The choice of external laboratory depended on the distributing laboratory’s preference.

Lessons learned from the analysis and reanalysis of discrepant results were documented in internal and shared reports between the participating laboratories and incorporated into routine practice as necessary and appropriate.

### Ownership of the isolates used in the EQA

Because the EQA scheme involves an international exchange of clinical isolates, in the EQA agreement the ownership of the collection is also described. All isolates remain the property of the province, state or country from which they were distributed. Isolates that are used in EQA panels may be retained by the participating laboratories for internal reference use, but the recipients may not share them with any other laboratory, nor use them for their own research purposes without the written consent of the laboratory from which they were distributed.

### Methods used by the EQA members

The methods used by the participating laboratories were mainly phenotypic tests although genotypic capsular typing was also used (Table 2). A general overview of the tests can be found described in previous literature^7,15,16^. The specific tests used by each participating laboratory are described in Table 2.

**Table 2:** Methods used by each participating EQA member.

EQA	Laboratory name	Method used
2005–2016B	SHLMPRL	Slide-agglutination using both Latex and standard type and factor antisera.
2005–2016B	SSI	Pneumotest Latex+Quellung reaction
2007A-2016B	NIPH	Quellung reaction for serotype identification using pool, group, type and factor sera.
2009A-2010B	PHE 1	Luminex antigen detection assay for serotypes 1, 3, 4, 5, 6A/C, 7F/A, 8, 9V, 14, Group 18, 19A, 19F and 23F with Slide agglutination for typing or subtyping if necessary, using standard type and factor antisera.
2011A-2016B	PHE 1	Slide agglutination both with Latex agglutination antisera and standard type and factor antisera
2014A- 2016B	PHE 2	Whole Genome Sequencing (WGS) + bioinformatic script was introduced as secondary method for testing and evaluation of the procedure.
2009A-2016B	THL	Primarily multiplex PCR modified from a protocol by the Centers for Disease Control and Prevention (CDC), if needed Quellung was used for further serotyping.
2015B-2016B	PHAS	Gel diffusion, Latex kit, and the Quellung reaction
2016-B	National Reference Lab. Israel	Capsular Sequence Typing (CST) PCR identification of all types. Quellung reaction for serotype identification using pool, group, type and factor sera.
2016-B	NRLBM	Type: Slide-agglutination, Subtype: Quellung reaction, Multi-Locus Sequence Typing (MLST): PCR-sequencing

### NIPH

Isolates were incubated on blood agar plates and serotyped by Quellung reaction using type-specific pneumococcal rabbit-antisera (SSI Diagnostica, Denmark). Optochin susceptibility and bile solubility tests were used for species identification, if necessary.

### SSI

#### Latex and Quellung

The pneumococci were identified by optochin susceptibility and bile solubility tests. All isolates were serotyped either by Quellung reaction alone or by the Pneumotest Latex kit (SSI Diagnostica, Denmark) combined with the Quellung reaction using type-specific pneumococcal rabbit-antisera as previously described (SSI Diagnostica, Denmark)^17^.

#### Latex agglutination test (ImmuLex™ Pneumotest kit, SSI Diagnostica, Denmark)

For determination of serogroup, the latex agglutination test was performed by using the ImmuLex™ test as described by the packaging inserts (SSI Diagnostica, Denmark). Briefly, isolates were cultured for 24 hours in Todd Hewitt broth (TH-broth, SSI Diagnostica, Denmark). Ten microlitres from this culture was mixed with specific antisera for observation of agglutination reactions.

#### The capsular reaction test (Quellung test)

For full serotyping, isolates were serotyped/confirmed by Quellung reaction alone (SSI Diagnostica, Denmark) combined with the Quellung reaction using type-specific pneumococcal rabbit-antisera as previously described (SSI Diagnostica, Denmark)^17^.

### PHE 1

From 2005–2010, isolates were grown on Columbia Blood Agar, a 1 µl loop of culture was used to inoculate a 2% deoxycholate solution in a PCR plate, this solution was then further diluted in PBS to a final dilution of ~1:2000 before being run in a Luminex antigen-capture assay using serotype specific monoclonal antibodies for serotypes 1, 3, 4, 5, 6 A/C, 6B, 7 F/A, 8, 9 V, 14, group 18, 19 A, 19 F and 23 F^18^. Any isolate not giving a definitive result in the assay was serotyped or subtyped using slide agglutination. After 2010 only the following slide agglutination method was performed due to the serotypes available in the Luminex assay becoming considerably less frequent in the population.

Isolates were grown for four hours or overnight in 5 ml MAST Todd Hewitt broth (PHE media services) at 35 °C with 5% CO_2_, centrifuged at 453x*g* for 30 min, and the supernatant removed, the cell pellet re-suspended in a small residual volume of broth and subjected to slide agglutination tests with latex antisera (ImmuLex™ Pneumotest kit) or standard factor sera (SSI Diagnostica, Denmark).

### PHE 2

From 2014 whole genome sequencing was introduced into the laboratory and an additional set of results was included with the phenotypic results while the technology was being developed for incorporation into routine use.

DNA extraction, whole genome sequencing and bioinformatic analysis for serotype determination was performed as described by Kapatai *et al*.^16^.

### SHLMPRL

Organisms were grown anaerobically on Columbia horse blood agar (Oxoid™) for 24 h. Slide agglutination was performed using Pansorbin cells absorbed with type specific pneumococcal anti-sera obtained from the Statens Serum Institute^19^.

### THL

The pneumococci were identified by the optochin susceptibility test, and if the result was inconclusive with the bile solubility test. Six sequential multiplex PCRs (mPCRs) were used for the deduction of serotypes. Quellung reaction was used to distinguish co-detected serotypes, for subtyping, and for serotyping if no serotype- or group-specific product was obtained in any of the sequential mPCRs^20^. The antisera for Quellung reaction were obtained from SSI Diagnostica, Denmark, and used according to the manufacturer’s recommendation.

### PHAS

The isolates were identified by the optochin susceptibility test, and if needed with the bile solubility test. All isolates were serotyped by gel diffusion using type specific pneumococcal anti-sera (SSI Diagnostica, Denmark), and some in addition by the Quellung reaction.

### NRLBM

The pneumococci were identified by optochin susceptibility. All isolates were serotyped by the Latex agglutination test (ImmuLex™ Pneumotest kit, SSI Diagnostica, Denmark combined with the Quellung reaction using type-specific pneumococcal rabbit-antisera as previously described (SSI Diagnostica, Denmark)^17^.

### National Reference Laboratory Israel

The isolates were grown on sheep blood agar for 18–24 h at 35 °C with 5% CO_2_. All isolates were typed in parallel by CST typing^21^, and Quellung (Neufeld antisera, SSI Diagnostica, Denmark). CST PCR sequences were analyzed on the BioNumerics 7.1 platform (Applied Maths) and compared with the global database. In case of discrepant results between the two methods, the Quellung result was reported.

### Questionnaire on Acceptability and Usefulness

In November 2016, a questionnaire regarding the acceptability and usefulness of participating in the EQA distributions was sent to all eight laboratories. The following questions were included in the questionnaire: 1. Is the frequency of EQA acceptable, or is it too frequent? 2. Is the workload for preparing and shipping panels acceptable? What is the limit? 3. Are the results useful to improve and maintain the performance of the laboratory? 4. Is the EQA useful for quality assurance purposes? 5. Is your laboratory accredited? If yes, what method or process is accredited, and according to which standard? 6. Is the EQA useful for accreditation purposes? 7. What could be changed or added?

## Results

For description and definition of different typing sera systems used, see the inserts from the producer (SSI Diagnostica, Denmark, www.ssidiagnostica.dk).

### EQA results

From June 2005 to September 2016, 22 EQA panels were distributed, each consisting of seven clinical pneumococcal isolates, i.e. including a total of 154 pneumococcal isolates. Panels were distributed twice a year, except for 2005 and 2006 when only one EQA panel was shipped; 2005A and 2006A, respectively (Table 3).

**Table 3:** EQA typing results from 2006–2016.

EQA	Number of participating laboratories	Tested serotype and number of correct identification Serotype (Number of correct identifications). Discrepant results which did not reach a final conclusion are in bold.							Fully correct answers	Final comments to EQA results. For details see results section.
2005-A	2 (SHLMPRL^*^, SSI)	9N (2)	23F (2)	17F (2)	23A (2)	1 (2)	22F (2)	18C (2)	Yes	First result from one lab showed a rough isolate instead of 17F. Retesting showed correct results.
2006-A	2 (SHLMPRL, SSI^*^)	23A (2)	19A (2)	14 (2)	3 (2)	38 (2)	31 (2)	15B (2)	Yes	
2007-A	3 (NIPH, SHLMPRL^*^, SSI)	11A (3)	4 (3)	18C (3)	6B (3)	7F (3)	9V (3)	27 (3)	Yes	
2007-B	3 (NIPH, SHLMPRL, SSI^*^)	33F (3)	24F (3)	11A (3)	22F (3)	37 (3)	19A (3)	15A (3)	Yes	
2008-A	3 (NIPH, SHLMPRL, SSI^*^,)	21 (3)	14 (3)	10A (3)	33F (3)	19F (3)	1 (3)	22F (3)	Yes	
2008-B	3 (NIPH, SHLMPRL^*^, SSI)	22F (3)	16F (3)	8 (3)	7F (3)	5 (3)	13 (3)	6A (3)	Yes	
2009-A	5 (NIPH, PHE, SHLMPRL, SSI^*^, THL)	15B (5)	9A (5)	23A (5)	16F (5)	38 (5)	4 (5)	9N (5)	Yes	
2009-B	4 (NIPH^*^, PHE, SHLMPRL, SSI)	19F (4)	35F (4)	6C (4)	6A (4)	33A (4)	24F (4)	15B (4)	Yes	One lab did not distinguish between 6A and 6C
2010-A	5 (NIPH, PHE, SHLMPRL^*^, SSI, THL)	31 (5)	35B (5)	12F (4) **12B (1)**	17F (5)	6B (5)	23B (5)	19A (5)	No	One laboratory had to retest the isolate before correct identification of 35B. Disagreement with 12 F/12B.
2010-B	5 (NIPH, PHE, SHLMPRL, SSI^*^, THL)	3 (5)	20 (5)	37 (5)	4 (5)	22F (5)	15A (5)	7F (5)	Yes	
2011-A	5 (NIPH, PHE, SHLMPRL, SSI, THL^*^)	7B (5)	1 (5)	6A (5)	8 (5)	34 (5)	11A (5)	14 (5)	Yes	
2011-B	5 (NIPH, PHE^*^, SHLMPRL, SSI, THL)	1 (5)	8 (5)	23B (5)	12F (5)	4 (5)	6C (5)	35B (5)	Yes	
2012-A	5 (NIPH^*^, PHE, SHLMPRL, SSI, THL)	9V (5)	19A (5)	23B (5)	6B (5)	7C (5)	19F (5)	7F (5)	Yes	1 lab had a problem with 9 g factor serum for 9 V. Retested and found correct serotype.
2012-B	5 (NIPH, PHE, SHLMPRL^*^, SSI, THL)	15A (5)	10A (5)	16F (5)	37 (5)	19A (5)	14 (5)	15C (4) **15B (1)**	No	See detailed explanation in results section
2013-A	5 (NIPH^*^, PHE, SHLMPRL, SSI, THL)	6B (5)	23A (5)	23B (5)	8 (5)	19F (5)	20 (5)	9N (5)	Yes	
2013-B	5 (NIPH, PHE, SHLMPRL, SSI, THL^*^)	6C (5)	22F (5)	3 (5)	18C (5)	9V (5)	11A (5)	35F (5)	Yes	
2014-A	5 (NIPH, PHE^*^, SHLMPRL, SSI, THL)	8 (5)	24F (5)	12F (5)	23B (5)	19A (5)	23A (5)	9N (5)	Yes	
2014-B	5 (NIPH, PHE, SHLMPRL, SSI^*^, THL)	9A (5)	11B (5)	37 (5)	31 (5)	29 **35B (3)** (2)	15B (5)	10B (5)	No	See explanation for disagreement in results.
2015-A	5 (NIPH, PHE, SHLMPRL^*^, SSI, THL)	21 (5)	9N (5)	3 (5)	16F (5)	22F (5)	23B (5)	7F (5)	Yes	
2015-A	6 (PHAS, NIPH^*^, PHE, SHLMPRL, SSI, THL)	10B (6)	15A (6)	6C (6)	9V (6)	23F (6)	24F (6)	17F (6)	Yes	
2016-A	6 (PHAS, NIPH^*^, 2xPHE, SHLMPRL, SSI, THL)	19F (6)	40 (2) **NT (4)**	6D (7)	20 (7)	15B (6)	23B (7)	9N (7)	No	See explanation for disagreement in results.
2016-B	8 (PHAS, National Reference Lab. Israel, NRLBM, NIPH, PHE^*^, SHLMPRL, SSI, THL)	33F (8)	25A (8)	10A (8)	3 (8	22F (8)	23A (8)	16F (8)	Yes	No factor sera available for serotype 25, were only able to identify to group level.

^*^Organizing laboratory.

**Figure 1:**
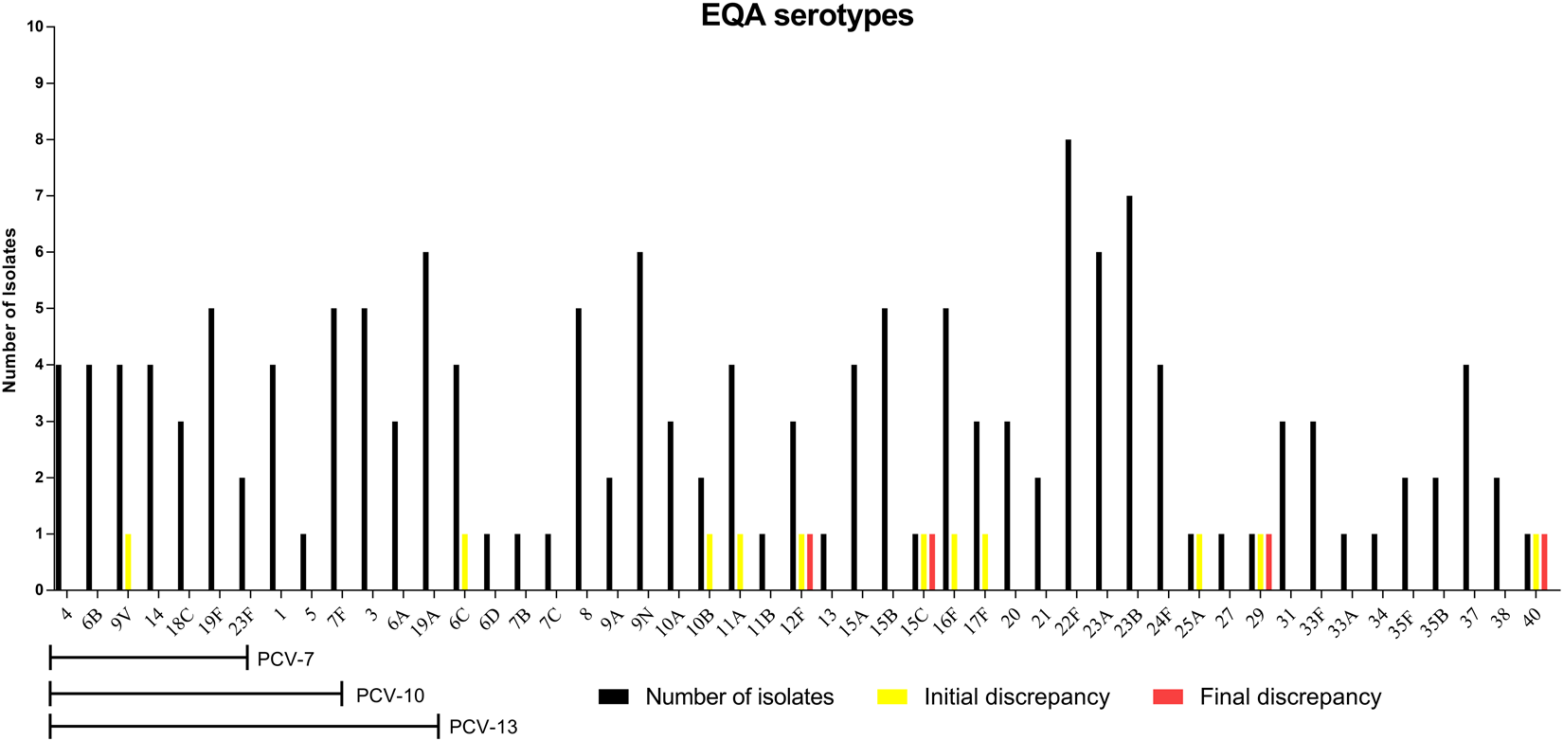
Serotypes included in the EQA, and the frequency of the tested isolates. The black bars below the X-axis present the serotypes included in the three different conjugated vaccines.

Overall, 49 distinct serotypes were included in the 22 panels (Fig. 1). All pneumococcal conjugate vaccine (PCV) serotypes, and all but one (serotype 2) pneumococcal polysaccharide vaccine (PPV) serotypes were included. Fully concordant serotyping results were observed for 14 EQA panels. Of the 154 isolates tested, 26 (17%) belonged to serotypes included in the 7-valent PCV, 36 isolates (23%) belonged to serotypes included in the 10-valent PCV, and 50 isolates (32%) belonged to serotypes included in the 13-valent PCV (Fig. 1). Serotype 22 F was most often included in the test panel, followed by 23B, 19 A, 9 N and 23 A. Around 40 pneumococcal serotypes were not included in any of the test panels. Combining all the tested isolates (n = 154), a discrepancy was found with 11 isolates (7.1%, 95% CI: 4% to 12%) at first test, and the discrepancy was not resolved in four distinct isolates (2.6%, 95% CI: 1% to 7%). The EQA panels showing a disagreement in the typing results are presented below.

### The EQA 2005-A

One laboratory found the 17 F isolate as a non-typeable isolate. However after retesting the isolate, it was found to be a 17 F isolate. The laboratory was not able to find the reason for the typing error in first test. This explanation was noted and accepted.

### The EQA 2009-B

EQA 2009-B showed a disagreement between the reference laboratories because one laboratory did not go further than the group level in serotype identification and did not determine the specific serotypes using factor sera. This limitation was noted and accepted.

### The EQA 2010-A

The first test showed a disagreement with the isolate 35B, however after retesting all laboratories agreed on the typing results of the isolate as 35B.

There was also a disagreement with isolate 12 F, with one laboratory serotyping this isolate as a 12B. The laboratory with the disagreement retyped the isolate several times, but was not able to confirm serotype 12 F. The laboratory changed the batch of its factor antisera but were however still not able to provide an explanation for the discrepancy. In 2017 the laboratory retested the isolate; both with phenotypic testing and molecular identification, and this time they were able to confirm the serotype of the isolate as 12 F. It was speculated that the original problem with the identification was due to difficulties with interpreting the agglutination reaction. The majority of other laboratories used the same lot of the 12 factor antisera.

### The EQA 2012-A

EQA 2012-A showed a preliminary disagreement for a serotype 9 V isolate, which by one laboratory was identified as a serotype 9 A, however after retesting the isolate at the laboratory, they agreed on serotype 9 V. The typing problem was referred to the problematic reading of a positive reaction of factor sera 9 g. This explanation was noted and accepted.

### The EQA 2012-B

EQA 2012-B showed a disagreement between the reference laboratories of identifying a serotype 15 C, which was serotyped as 15B by one reference laboratory. The consensus result was 15 C. However, laboratories did report that cross-reactions sometimes did occur with the group 15 factor sera and differentiation between 15B and 15 C was proven quite difficult. The reference laboratory identifying the 15B, found that with individual factor sera interpretations in Quellung, the reactions with factor sera 15b and 15e were positive, 15c was negative, and 15 h was interpreted as a weak positive possible cross-reaction (SSI Diagnostica, Denmark, insert for factor sera). At the time, it was described that serotypes 15B and 15 C may interconvert through a reversible event, which was one explanation for why the antisera reactions were not straightforward to interpret. The explanation was based on published data on serotype 15B versus 15 C^22–24^. One laboratory noted that they had experience in identifying both 15B and 15 C from one culture/plate due to different morphologies, and to identify the two types from the same cultures on repeated typing following freezing. They had however not observed problems with cross-reactions of factor sera, and the reactions were usually easy to interpret. The laboratory reports 15B and 15 C as distinct serotypes in their internal laboratory reports, but for surveillance reports they usually combine 15B/C as a group. Discrepancies between 15C and 15B were also reported in for the Circumpolar pneumococcal EQA^10^.

For serotype 10 A and 16 F in the panel, one laboratory did not type further than to group level, therefore the identification result was group 10 and 16.

### The EQA 2014-B

EQA 2014-B showed a disagreement between the reference laboratories on a serotype 29 versus 35B isolate. The organizing laboratory determined it as type 29 while three other laboratories found it as type 35B. One laboratory could not differentiate between serotype 29 or 35.

The organizing laboratory found a reaction in pool G and for 29, 35 and 42. When using the factor-sera following reactions were observed: 35a (negative), 35b (negative), 35c (+++), 29b (+++), 42 (negative). Due to the missing reaction with factor-sera 35a, the isolate was not of type 35 but rather 29. One laboratory identified the serotype 35B directly through a multiplex PCR (mPCR) instead of using the Quellung. The primers used in the mPCR amplify the *wcrH* locus^25^. Aligning these primers with the serotype 29 reference sequence *S. pneumoniae* isolate 34373 (serotype 29) CR931694 did not show significant similarity, and there had not been any previous reports about discrepancy between mPCR and Quellung results for serotype 35B isolates.

Another laboratory concluded that it was serotype 35B, based on reactions in 35b, 35c, 29b, and 42a, as well as 29 and 35. The laboratory did not use the 35a serum; however, after retesting with 35a, they still interpreted the reactions as ambiguous. The packet insert (SSI Diagnostica, Denmark) stated a possible cross-reaction between type 29 and 35B. Although a reaction in 35a is needed for serotype 35B, it is not evident from the packet insert that a negative reaction in 35a is used to identify serotype 29. The laboratory also performed CST-typing (*wzh* gene sequencing)^21^. The result, with 100% match, indicated capsule type 15C-01, with corresponding serotypes 17 A, 35B and 35 C. There was no mention of serotype 29. This result was confirmed by another participating laboratory.

In conclusion the isolate was found to genetically resemble a serotype 35B, however it was concluded that phenotypically it was a serotype 29. The isolate was tested by SSI Diagnostica (Denmark), the company producing the typing sera, and they confirm it to be phenotypically serotype 29. Discrepancies between serotype 29 and 35B have been reported by the Circumpolar EQA as well^10^. In this study the discrepancy was explained by the presence of reaction with factor serum 29b in the antigenic formula of both serotypes.

### The EQA 2016-A

EQA 2016-A showed a disagreement between the reference laboratories for two isolates (serotype 19 F and 40).

Serotype 19 F isolate: All results were concordant by conventional phenotypic methods, although one laboratory found that the isolate provided a strong reaction with 19b latex sera but also some reaction with 19c latex sera. With WGS the isolate identified as genotype 19 A by the current WGS pipeline, however it appeared to have an unusual sequence properties. Another laboratory mentioned that serotype 19 F isolate only produced the internal control band in the mPCR and was serotyped through Quellung.

Regarding the serotype 40 isolate, one laboratory found the identification challenging. The isolate did not react in the group-7 serum, but did react with the factor-serum. A second laboratory found that the serotype 40 isolate caused some problems as it reacted ambiguously. Even the bile-solubility test was inconclusive. It provided a weak response in the latex pool C and could possibly be a serotype 24, but this was not a definitive result. A third laboratory mentioned that the isolate showed a reaction with pool C but further typing was not possible due to auto-agglutination. The laboratory mentioned that the isolate failed the WGS serotyping pipeline with CPS operon coverage to nearest serotype just under the 90% cut-off. It was most similar to 7B, 7 C, 40. It also had a new MLST type ST11674, due to a novel *spi* allele. One of the laboratories mentioned that the serotype 40 isolate was mPCR positive for *wzg* (=*cpsA*, internal positive control) and produced an amplicon that co-detects serotypes 7 C, 7B, and 40 through the *wcwL* gene. To distinguish co-detected serotypes the Quellung reaction was used. The reaction for group 7 serum was negative and serotype 40 serum was positive, thus the laboratory result was serotype 40. The isolate also reacts positively with factor serum 7 f.

### Acceptability and usefulness of the EQA

The questionnaire results showed that all laboratories found that the distribution frequency of two EQA panels a year was acceptable (Question 1), although one laboratory mentioned that one EQA panel a year would be sufficient. Another laboratory stated that more than two EQA panels a year would be too many. In general the workload for preparing and shipping seven isolates to eight laboratories (question 2) was found acceptable, although it was stated by several laboratories that it was close to the limit. One laboratory mentioned the possibility of using an external provider to supply the test samples as an alternative to the laboratories doing it themselves and thereby possibly increase efficiency and reduce the cost. Three laboratories had not yet performed the task of sending the test panels.

All laboratories found that the EQA results (Question 3) were useful for improving and maintaining the performance of the laboratory. Several laboratories found that, using the results, they were able to improve their identification procedure. It was mentioned by one laboratory, that some panel results generated an interesting discussion regarding comparison and the use of different typing methods particularly between genetic and phenotypic methods.

All laboratories agreed that the EQA was useful for their quality assurance (Question 4). For seven laboratories the pneumococcal serotyping is accredited (Question 5), while one laboratory is in the process of obtaining an accreditation. For three laboratories the accreditation was specific for the serotyping of pneumococcal isolates. The standards included SWEDAC ISO17025, NS-EN ISO/IEC17025, DANAK ISO17025, UKAS ISO15189:2012, SFS-EN ISO/IEC17025:2005, ISO1518, ISO15189.

All laboratories found that the EQA was useful for their accreditation. One laboratory was in the process of obtaining their accreditation, and would use the EQA for this purpose (Question 6). One laboratory mentioned that they used a separate EQA as part of their accreditation.

Several changes to the EQA scheme were suggested (Question 7): to include molecular characterisation to present predicted serotype or the MLST type, to share the laboratory test protocols to enable standardisation of all identification protocols in the participating laboratories, and to add more isolates to the panel to cover more of the serotypes they received.

## Discussion

The purpose of an EQA is to compare the results and interpretations of laboratory analyses. This serves as a quality assurance for the participating laboratory, provision of early warning for systematic problems associated with kits or operations; provision of objective evidence of testing quality; indication of areas that require improvement and to identify training needs, and to improve comparability and harmonized reporting between laboratories despite the use of different laboratory methods. EQAs are used in many different settings^11,13,26^ both to provide a quality statement^9,10,26^, but also to help guide the reference laboratories in providing the appropriate laboratory tests^9,10,12^. This paper describes an EQA scheme for *S. pneumoniae* serotype identification arranged between national reference laboratories that started in June 2005 and is still on-going. The EQA demonstrate a high concordance of serotype results between the participating laboratories, although different serotype identification methods are used. Other pneumococcal EQA studies with different setup, test interval and number of isolates have been presented although with variable results regarding the concordance of serotype results^9,10,14^.

The typing procedures used at the participating laboratories were different (Table 2), although the Quellung test (six laboratories) and slide agglutination test (three laboratories) were used as the final serotype identification procedure most of the laboratories (Table 2). The majority of the typing discrepancies were due to inability to serotype further than the group level, or problems with interpretation of the reactions when using factor sera. In general we found that although different identification procedures were used between the laboratories, the discrepancies were few (Fig. 1). Of the 154 isolates tested over the years, only a 7.1% discrepancy was observed after the initial testing, and after the final testing this rate declined to 2.6%. This level of discrepancy is similar to the around 5% observed in other pneumococcal studies on validation of laboratories pneumococcal serotyping^9,10,14^. However, a few isolates did provide typing disagreements, which were at the time not possible to resolve satisfactorily. During the study period, four isolates did not reach a final identification agreement between all participant laboratories (Table 3). Discrepancies with no satisfactory solution have also been observed by Reasonover *et al*.^10^, in which they reported 4 isolates with no final agreement on the typing result. Reasonover *et al*.^10^ also found problems with the identification of group 15 isolates and between serogroup 29 and serotype 35B, as also seen in this study. Also other EQA programs describe discrepancies for which there were no explanations to be found^10,14^.

One laboratory has started to include WGS of pneumococcal isolates, however the use of WGS and other molecular based methods compared to phenotypic methods does highlight potential differences, which need to be discussed before molecular methods can replace phenotypic methods. The laboratory using the WGS method has tried to match the phenotypic methods as far as possible. However genotypic methods may sometimes pick up new genotypes, which may only later be described phenotypically or may indeed correspond to existing phenotypic serotypes despite being genetically different^16^. Therefore a discrepancy may not be a mistake but rather a new variant and it will take more time to investigate such issues, e.g. the 29/35B discrepancy observed in EQA 2014-B^16^. Using phenotypic methods versus molecular methods will therefore give disagreements between reference laboratories, particular with regards to the number of isolates defined as non-typeable. These are problems that are also seen by other groups working with pneumococcal typing methods^7,16^.

Because many laboratories perform their pneumococcal identification scheme in relation to monitoring the effect of introduced pneumococcal vaccines^6,7,27^, it is essential to provide an accurate serotype identification. It is therefore important to find a procedure in the future that incorporates the benefit from both capsular gene-based typing and capsular phenotyping. One possible future identification protocol could be the protocol presented by Kapatai *et al*.^16^ where a WGS method is used for capsular genotyping, and based on the WGS results from a specific isolate additional phenotypic tests are performed if necessary. For example when a molecular serotype is not obtained, or when the appearance of colonies indicates a lack of capsule production (rough/small appearance on culture plates). Another study has also suggested the use of molecular methods for screening followed by phenotypic methods for examinations^28^. Including WGS in the identification procedure will also provide easy determination of the MLST type and presence of resistance genes^15^. Because there are still unsolved differences between capsular gene based methods and capsular phenotyping methods^16,28^, EQAs will probably experience a higher number of discrepancies if EQA programmes involve laboratories using a mixture of these methods, compared to an EQA involving only laboratories using phenotypic methods. It is therefore not yet recommended to directly compare the outcome from these two typing procedures in an EQA.

In this study, we have only presented data based on serotyping of confirmed pneumococcal isolates. However, a growing problem for many microbiological laboratories is the separation of *S. pneumoniae* from other species within the viridans group^29^. For example a method such as optochin susceptibility has recently been recommended not to be used for confirming *S. pneumoniae* due to increasing number of optochin resistance pneumococcal isolates^30^. It is therefore essential that isolates used in an EQA are properly identified as *S. pneumoniae*.

Serotyping of pneumococci is performed by accredited methods in seven of the eight participating reference laboratories. Responses to the questionnaire on acceptability and utility indicated that the EQA scheme is important for continuous quality assurance and improvement, and for achieving and maintaining accreditation of laboratory procedures. The EQA scheme is run by collaboration between laboratories, and does not receive specific funding. Although this is currently described as acceptable by the participants, an increased work-load by including more laboratories, or additional laboratory procedures, might tip this into unacceptable.

Lessons learnt from this EQA since 2005 can be formulated into good advice for laboratories that are planning to set up an EQA, as listed below:

1. Prepare an EQA contract describing the agreement between the laboratories, including the ownership of the isolates. In the described EQA all participants have signed an EQA contract. By preparing an EQA contract, describing in detail what is required from each participant will ensure that all participants know what to expect when discrepancies are found and discussed. Because this EQA is dependent on participants in turn distributing seven clinical isolates from their own collection, it is important that the participants are aware of the limitations for use of the isolates, according to the contract.
2. Each participant/prospective participant laboratory need to ensure the customs laws in a proposed participant country allow for shipment of live microorganisms. At one time a laboratory tried to participate in this EQA, but had to leave it because their national customs laws would not allow the import of the isolates.
3. Although relatively inexpensive, some cost has to be expected when participating in an EQA. In this EQA there are particular costs associated with being the panel sending laboratory for staff time, materials and shipping of the seven isolates to the other participants, although this task changes hands each time. Also, a cost has to be expected for the simple procedure of identification of the seven isolates, particularly if discrepancies are found and repeats are necessary. In this EQA everything is based on voluntary participation under guidelines from a signed contract (rules). However it also has to be considered that there are private companies e.g., UKNEQAS (www.ukneqas.org.uk) that provide services within this field. Cost/benefit therefore has to be considered when choosing to participate in an EQA.
4. Consideration should be made as to the maximum number of participating laboratories. In this EQA, it was found that the limitation of participants is around eight to ten laboratories before it will become too labour intensive due to the large number of replicates of isolates to be prepared for sending.
5. Agree on the type and number of isolates that are to be included in the EQA. In this EQA we use every day clinical isolates that the administrating laboratory choose. Seven isolates are close to the maximum as this means that a minimum of 56 isolate slopes (and usually several extra as spares) need to be prepared for eight participating laboratories. In this EQA, it is the distributing participant that decides the serotypes of isolates to be sent. Again here it is important to evaluate what the needs for a participant are. If it is quality assurance of routine test procedures for common clinical isolates, then this EQA will be suitable. However, if it is identification procedures for other purposes, such as developing new methods, then it has to be considered if another type of EQA is needed, where for example more specific or rare isolates are included.
6. Consider how often the EQA should be performed each year. We have found that two EQAs a year is a good compromise between practicality and regularity. Some laboratories mentioned that one EQA a year would be enough for them to cover their needs. More than two EQA rounds a year is too many.

## Conclusion

For the laboratories participating in the EQA the general conclusion is that the EQA participation helps the individual laboratory to keep their typing procedures at a high standard, ensuring that the output can be compared to the pneumococcal typing output of other countries, and where appropriate, highlighting when adjustments and improvements are needed. It furthermore shows that pneumococcal typing data presented from the participating laboratories can be compared in surveillance studies, with the assurance that the different typing procedures used in the different countries provide a consistent output, and therefore should not introduce a bias factor to be considered when interpreting the data. The EQA is also helpful when introducing new technologies, adding validation data and also showing limitations of both genotypic and phenotypic methods that need to be taken into account when interpreting data that is obtained from both methods. Continuation of the presented EQA program is scheduled.
